# Infection Risk in Older Kidney Transplant Recipients: An Analysis in the Era of Expanded Age Limits

**DOI:** 10.3389/ti.2025.15594

**Published:** 2025-11-12

**Authors:** Marina Fayos, Laura Corbella, Isabel Rodriguez-Goncer, Hernando Trujillo, Francisco López-Medrano, Esther González, Ana Hernández, Tamara Ruiz-Merlo, Rafael San-Juan, Natalia Redondo, Amado Andrés, José María Aguado, Mario Fernández-Ruiz

**Affiliations:** 1 Unit of Infectious Diseases, Hospital Universitario “12 de Octubre”, Instituto de Investigación Sanitaria Hospital “12 de Octubre” (imas12), Madrid, Spain; 2 Centro de Investigación Biomédica en Red de Enfermedades Infecciosas (CIBERINFEC), Instituto de Salud Carlos III, Madrid, Spain; 3 Department of Medicine, School of Medicine, Universidad Complutense, Madrid, Spain; 4 Department of Nephrology, Hospital Universitario “12 de Octubre”, Instituto de Investigación Sanitaria Hospital “12 de Octubre” (imas12), Madrid, Spain

**Keywords:** kidney transplantation, infection, age, older adult recipient, incidence

## Abstract

The expansion of eligibility criteria has led to an increase in the age at kidney transplantation (KT), with consequences on the infection risk. We performed a prospective single-center cohort study of 712 patients undergoing KT between 2014 and 2022. Recipient age (median: 56.6 years [interquartile range: 43.2–68.5]) was analyzed by 10-year strata and dichotomized by thresholds (≥60, ≥70, ≥75 and ≥80). Univariable and multivariable regression models were constructed to assess the incidence of overall, bacterial and opportunistic post-transplant infection. In unadjusted analyses, each 10-year-increase was associated with overall (subdistribution hazard ratio [SHR]: 1.18; 95% confidence interval [CI]: 1.11–1.26), bacterial (SHR: 1.17; 95% CI: 1.09–1.26) and opportunistic infection (SHR: 1.26; 96% CI: 1.13–1.40). All groups >50 had an increased risk of infection. After multivariable adjustment, this association remained significant for overall (adjusted SHR [aSHR] per 10-year-increase: 1.09; 95% CI: 1.02–1.18) and bacterial infection (aSHR per 10-year-increase: 1.09; 95% CI: 1.00–1.18). Recipients ≥60 exhibited higher risk of overall infection (aSHR: 1.25; 95% CI: 1.00–1.54), and recipients ≥70 higher risk of opportunistic infection (aSHR: 1.54; 95% CI: 1.02–2.32). The incidence of infection was not significantly higher for patients ≥80 years. In conclusion, infection risk after KT increases with age, notably beyond 60 years.

## Graphical Abstract

**Figure:**
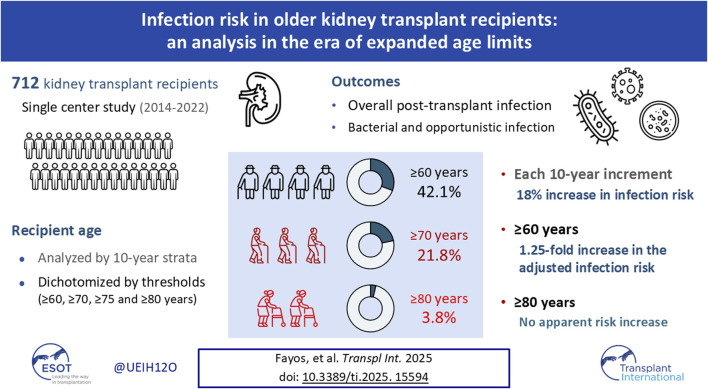

## Introduction

Kidney transplantation (KT) is the best treatment option for end-stage renal disease (ESRD) [1], as it offers significant benefits in terms of life expectancy and health-related quality of life. The proportion of older adult patients on the waiting list has steadily risen over recent decades, paralleling an increase in the number of procedures performed in this demographic [1, 2]. In Europe, the average age at transplantation has increased by 10 years during the past 20 years, whereas the percentage of US patients on the waiting list aged 65–74 years surged from 2% in the 1990s to over 10% by 2012 [1]. A growing number of studies demonstrate that KT is a feasible option for selected older patients, with a survival benefit compared with remaining on dialysis [3–9]. Fostered by these favorable experiences, the expansion of the limit age for KT seems to have no limits. For instance, our group reported acceptable patient and graft outcomes for recipients ≥75 years that received a graft from similarly aged deceased donors [10], whereas a registry-based analysis found no significant differences in death-censored graft survival between recipients ≥80 years and those aged 60–69 years [11].

Aging is often accompanied by complex medical conditions, such as cumulative comorbidity burden, sarcopenia, frailty and cognitive impairment, which can act as barriers to KT [12–14]. Thymic involution and epigenetic alterations contribute to the age-related dysfunction of innate and adaptive immune responses [15]. This process of immunosenescence is linked to a chronic low-grade pro-inflammatory state or inflammaging [16], leading to an increased susceptibility to complications due to over-immunosuppression [17, 18].

The 2020 Kidney Disease: Improving Global Outcomes (KDIGO) guidelines states that advanced age alone should not be considered as an contraindication to KT [19], in line with some national recommendations [20, 21]. The eligibility criteria should balance the expected benefit against the risk of associated complications, such as post-transplant infection. Death with a functioning graft (DWFG) is the most common cause of graft loss in the older KT population [22], and infection remains as one of the leading causes of death [11]. Unfortunately, the independent impact of age on the risk of infection has not been yet explored in depth. Indeed, a systematic review revealed the underreporting of post-transplant complications, including infection, in the studies that have investigated the outcomes of older adults [23].

In the current scenario in which old age no longer precludes ESRD patients from undergoing KT, it is essential to understand the relative contribution of this factor to the risk of infection. To this end, we have analyzed a large single-center cohort historically characterized by the use of kidneys from aged donors in older patients [10]. Since the literature lacks a consensus definition for the evolving concept of “older KT recipient,” recipient age has been analyzed as a continuous variable as well as dichotomized using various cut-off values, to ultimately determine the threshold at which the risk of infection increases.

## Materials and Methods

### Study Population and Setting

We performed a prospective observational cohort study that included all consecutive adult ESRD patients (≥18 years) that underwent KT at the University Hospital “12 de Octubre” (Madrid, Spain) between November 2014 and December 2022. We also included combined KT procedures (i.e., pancreas-kidney, liver-kidney and heart-kidney). Patients experiencing primary graft non-function, death or graft loss within the first week were excluded. The study was performed in accordance with the ethical standards laid down in the Declarations of Helsinki and Istanbul. The local Ethics Committee approved the study protocol (CEIC number: 14/030) and written informed consent was obtained from all participants at the time of recruitment to the institutional cohort.

### Study Design

The study cohort has been described elsewhere [24–26]. Participants were enrolled at the time of transplantation and followed-up for at least 1 year or, alternatively, until graft loss or death if occurred earlier. Patients were seen regularly at the outpatient clinic at scheduled follow-up visits or whenever clinically indicated. Demographics, clinical, laboratory and microbiological variables and post-transplant events were prospectively collected in a standardized case report form.

The *primary study outcome* was the occurrence of overall post-transplant infection, with recipient age at transplantation as the independent variable of interest. Bacterial and opportunistic infection were considered s*econdary outcomes*. As an exploratory approach to immunosenescence, we analyzed the association between recipient age and peripheral blood lymphocyte subpopulation counts (CD3^+^, CD4^+^ and CD8^+^ T-cells) and the CD4+/CD8+ ratio throughout the first post-transplant year in patients that did induction therapy with T-cell-depleting agents such as anti-thymocyte globulin (ATG).

### Immunosuppression and Prophylaxis Regimens

All recipients of organs from donors after circulatory death underwent induction therapy with ATG (either Thymoglobulin® at a dose of 1–1.5 mg/kg/day for 5–7 days or Grafalon® at a dose of 3 mg/kg/day for 3 days), with delayed introduction of a calcineurin inhibitor (CNI) from day 6. Recipients at high immunological risk also received ATG induction with early CNI initiation from post-transplant day 0. Basiliximab (20 mg on days 0 and 4) with delayed CNI introduction from day 5 was reserved to patients at high risk for CNI-related nephrotoxicity (i.e., older age or comorbidities). Maintenance immunosuppression consisted of tacrolimus (0.1 mg/kg daily, adjusted to a target trough level of 10–15 ng/mL during the first month and 5–10 ng/mL thereafter); mycophenolate mofetil (1 g twice daily) or enteric-coated mycophenolate sodium (360 mg twice daily); and prednisone (1 mg/kg daily with progressive tapering). Conversion to mammalian target of rapamycin (mTOR) inhibitor-based regimens with reduced-dose tacrolimus (target trough level of 3–6 ng/mL) was performed on an individual basis for recipients experiencing severe CNI-related adverse effects, difficult-to-treat cytomegalovirus (CMV) infection, or malignancy.

All patients received preoperatively a single dose of intravenous (IV) cefazolin (or ciprofloxacin in case of ß-lactam hypersensitivity). Prophylaxis for *Pneumocystis jirovecii* pneumonia was administered with trimethoprim-sulfamethoxazole (160/800 mg three times weekly) or monthly intravenous pentamidine for 9 months. In patients at high-risk for CMV infection, universal prophylaxis with oral valganciclovir (900 mg daily) was given for 3 (seropositive recipients [R+] that received ATG induction) or 6 months (serology mismatch [donor positive/recipient negative (D+/R-)]). Intermediate-risk patients (R+ without ATG induction) were managed by polymerase chain reaction (PCR)-guided pre-emptive therapy, and IV ganciclovir (5 mg/kg/12 h) or oral valganciclovir (900 mg/12 h) for at least 2 weeks was initiated in the presence of high level or rapidly increasing CMV DNAemia. Valganciclovir doses were adjusted according to renal function as necessary [27].

### Study Definitions

The diagnosis of post-transplant infection was established by at least one of the following criteria: a) positive culture of an unequivocally pathogenic microorganism from any sample; b) isolation of any microorganism from a sample obtained under sterile conditions; c) isolation of a potentially pathogenic microorganism from any sample obtained from sterile or non-sterile sites (where urine was always considered a non-sterile specimen irrespective of whether the sample had been collected by the midstream clean-catch technique or catheterization), accompanied by clinical manifestations compatible with the different infectious syndromes (e.g., fever, chills, lumbar pain, graft pain and/or irritative voiding symptoms in the case of pyelonephritis); and/or d) clinical data suggestive of infection without microbiological isolation and complete resolution under empirical antimicrobial treatment. Episodes of asymptomatic bacteriuria, cystitis, asymptomatic CMV DNAemia and low-level BK polyomavirus DNAemia were excluded. Opportunistic infection included CMV disease, infections due to intracellular bacteria (e.g., *Listeria monocytogenes* or mycobacteria), other herpesviruses (herpes simplex virus and varicella-zoster virus), yeasts (*Candida* spp. and *Cryptococcus* spp.), molds, *P. jirovecii* and parasites (*Cryptosporidium*, *Toxoplasma gondii* and *Leishmania* spp.) [28]. Proven or probable invasive fungal disease was defined based on the criteria proposed by the European Organization for Research and Treatment of Cancer and the Mycoses Study Group [29]. CMV disease comprised viral syndrome or end-organ disease, as detailed in Supplementary Methods [30]. Further definitions are also provided as Supplementary Material.

### Immune Evaluation

Peripheral blood lymphocyte subpopulation (CD3^+^, CD4^+^ and CD8^+^ T-cells) counts were measured at post-transplant months 1, 3, 6 and 12 by means of an automated multicolor flow cytometry system (BD Multitest™ six-color TBNK reagent with acquisition on the BD FACSCanto II instrument using BD FACSCanto clinical software, all from BD Biosciences, San Jose, CA).

### Statistical Analysis

Quantitative data were summarized by the mean ± standard deviation (SD) or the median with interquartile range (IQR). Qualitative variables were expressed by absolute and relative frequencies. Categorical variables were compared using the χ^2^ test. Student’s t-test or U Mann-Whitney test were applied for normally distributed continuous variables, and the Kruskal-Wallis test was used for comparing T-cell counts across increasing age groups.

Univariable and multivariable Fine and Gray’s competing risk regression models were fitted to assess the impact of recipient age at transplantation on the occurrence of overall, bacterial and opportunistic infection, with death from any cause as the competing event. The variable “recipient age” was analyzed in different ways: as a continuous manner by 10-year strata (age groups), and dichotomized according to the cut-off values used in the literature to define “older KT recipient” (≥60, ≥70, ≥75 and ≥80 years) [23]. We selected all demographic and clinical factors with a *P*-value < 0.05 in the univariable analysis to be entered in the multivariable models as independent (explanatory) variables. Collinearity was assessed by the variance inflation factor (VIF), with values <2 considered as acceptable. Since recipient age was our main variable of interest, it was always retained in the model in the presence of significant collinearity with other factors (e.g., donor age).

Associations were expressed as subdistribution hazard ratios (SHRs) with 95% confidence intervals (CIs). Statistical analysis was performed with SPSS version 29.0.1.0 (IBM Corp., Armonk, NY) and StataNow version 19.5 (StataCorp College Station, TX).

## Results

### Study Population and Outcomes

We included 712 KT recipients, whose demographics and clinical features are shown in Table 1. Median and mean age at transplantation were 56.6 (IQR: 43.2–68.5) and 55.4 ± 15.6 years, respectively. According to pre-established age thresholds, 300 (42.1%) patients were ≥60 years, 155 (21.8%) were ≥70 years, 84 (11.8%) were ≥75 years, and 27 (3.8%) were ≥80 years.

**TABLE 1:** Demographics and clinical characteristics of the study cohort (n = 712).

Variable	
Age at transplantation, years [median (IQR)]	56.6 (43.2–68.5)
Male gender [n (%)]	476 (66.9)
Prior or current smoking history [n (%)]	269 (37.8)
BMI at transplantation, Kg/m^2^ [mean ± SD]^a^	25.8 ± 6.4
Pre-transplant conditions [n (%)]	
Hypertension	591 (83.0)
Diabetes mellitus	204 (28.7)
Non-coronary heart disease	99 (13.9)
Coronary heart disease	79 (11.1)
Chronic pulmonary disease	77 (10.8)
Solid organ cancer	71 (10.0)
Cerebrovascular disease	46 (6.5)
Lower limb peripheral arterial disease	47 (6.6)
Previous KT [n (%)]	116 (16.3)
Underlying end-stage renal disease [n (%)]	
Glomerulonephritis	151 (21.2)
Diabetic nephropathy	141 (19.8)
Polycystic kidney disease	98 (13.8)
Nephroangiosclerosis	69 (9.7)
Chronic interstitial nephropathy	35 (4.9)
Loss of renal mass and hyperfiltration injury	19 (2.7)
Reflux nephropathy	22 (3.1)
Lupus nephropathy	25 (3.5)
Congenital nephropathy	30 (4.2)
Unknown	78 (11.0)
Other	44 (6.1)
CMV serostatus [n (%)]	
D+/R+	502 (70.5)
D-/R+	104 (14.6)
D+/R-	81 (11.4)
D unknown/R+	6 (0.8)
D-/R-	19 (2.7)
Positive EBV serostatus [n (%)]	653 (91.7)
Positive HCV serostatus [n (%)]	48 (6.7)
Positive HBsAg status [n (%)]	20 (2.8)
Positive HIV serostatus [n (%)]	5 (0.7)
Pre-transplant renal replacement therapy [n (%)]	626 (87.9)
Hemodialysis	499/626 (70.1)
Continuous ambulatory peritoneal dialysis	127/626 (17.8)
Time on dialysis, days [median (IQR)]	707 (375–1,383)
Type of transplantation [n (%)]	
Single kidney	666 (93.5)
Double kidney	3 (0.4)
Simultaneous pancreas-kidney	32 (4.5)
Pancreas after kidney	2 (0.3)
Combined liver-kidney	7 (1.0)
Combined heart-kidney	2 (0.4)
Age of donor, years [median (IQR)]	54.0 (44.0–66.0)
Type of donor [n (%)]	
DBD donor	470 (66.0)
Uncontrolled DCD donor (Maastricht categories 1–2)	68 (9.6)
Controlled DCD donor (Maastricht categories 3–4)	58 (8.1)
Living donor	116 (16.3)
Cold ischemia time, hours [mean ± SD]	15.7 ± 7.5
Number of HLA mismatches [median (IQR)]	4.0 (3.0–5.0)
Induction therapy [n (%)]	
Antithymocyte globulin	307 (43.1)
Basiliximab	323 (45.4)
None	81 (11.4)
Primary immunosuppression regimen [n (%)]	
Prednisone, tacrolimus and MMF/MPS	667 (93.7)
Prednisone, tacrolimus and azathioprine	29 (4.1)
Prednisone, tacrolimus and mTOR inhibitor	13 (1.8)
CMV prevention strategy [n (%)]	
Antiviral prophylaxis	375 (52.7)
Duration of prophylaxis, days [median (IQR)]	101 (90–169)
Preemptive therapy	335 (47.1)
Post-transplant complications at 1 year [n (%)]	
Delayed graft function	290 (40.7)
Surgical reintervention within the first month	114 (16.0)
Renal artery stenosis	89 (12.5)
New-onset diabetes	97 (13.6)
Development of *de novo* DSA	44 (6.2)
Biopsy-proven acute graft rejection	67 (9.4)
>1 episode	13 (1.8)
Time from transplantation, days [median (IQR)]	118 (19.8–283.5)
T-cell-mediated rejection	35 (4.9)
Borderline T-cell-mediated rejection	24 (3.4)
Antibody-mediated rejection	17 (2.4)

BMI, body mass index; CMV, cytomegalovirus; D, donor; DBD, donation after brain death; DCD, donation after circulatory death; DSA, donor-specific antibody; EBV, Epstein-Barr virus; HLA, human leukocyte antigen; HBsAg, hepatitis B virus surface antigen; HCV, hepatitis C virus; HIV, human immunodeficiency virus; IQR, interquartile range; KT, kidney transplantation; MMF/MPS, mycophenolate mofetil/enteric-coated mycophenolate sodium; mTOR, mammalian target of rapamycin; R, recipient; SD, standard deviation.

^a^ Data on BMI was not available for 115 patients.

The distribution of pre-transplant comorbidities and transplant-related variables in the different age strata is detailed in Supplementary Table S1. The prevalence of some conditions (non-coronary heart disease or cerebrovascular disease) and pre-transplant solid organ malignancy showed a clear gradient across increasing age strata. This association was less evident for other comorbidities, which peaked either at 60–70 (coronary heart disease, chronic pulmonary disease and peripheral arterial disease) or 70–80 years (hypertension and diabetes). The proportion of patients at the high-risk category for CMV infection (D+/R-) decreased with age. Regarding the type of donor, the proportion of living donation and donation after circulatory death also decreased with age.

After a median follow-up period of 728 days (IQR: 544.8–1,071), graft loss and all-cause death occurred in 33 (4.6%) and 68 patients (9.6%), respectively. One- and two-year survival rates were 95.3% and 91.9%, whereas the corresponding estimates for death-censored graft survival were 96.9% and 94.7%, respectively.

### Post-Transplant Infection

There were 1,088 distinct episodes of infection in the entire cohort. As shown in Supplementary Table S2, most common clinical syndromes were acute pyelonephritis (306 out of 1,088 episodes [28.1%]), viral syndrome (115 [10.5%]), digestive tract infection (114 [10.5%]), upper respiratory tract infection (95 [8.7%]), pneumonia (91 [8.4%]) and skin and soft tissue infection (90 [8.3%]). Overall, 155 episodes (14.2%) were associated to bacteriemia.

The predominant bacterial pathogens were *Escherichia coli* (136 out of 1,088 episodes [12.5%]), *Klebsiella pneumoniae* (92 [8.5%]), *Pseudomonas aeruginosa* (55 [5.1%]), *Enterococcus* spp (50 [4.6%]) and *Clostridioides difficile* (44 [4.0%]). Regarding antimicrobial susceptibility profiles, 77.2% (71/92) and 22.1% (30/136) of *K. pneumoniae* and *E. coli* isolates, respectively, were extended-spectrum β-lactamase (ESBL) producers, whereas one third of *P. aeruginosa* isolates (29.1% [16/55]) exhibited a multidrug-resistant phenotype (i.e., non-susceptibility to at least one agent in ≥3 antibiotic classes). Herpesviruses accounted for 163 episodes of infection (14.9%), with predominance of CMV (102 [9.4%]).

Within the subgroup of microbiologically documented episodes of urinary tract infection, *E. coli* (40.7% [116/285]), *K. pneumoniae* (28.4% [81/285]) and *P. aeruginosa* (8.8% [25/285]) were the most commonly isolated uropathogens (Supplementary Figure S1).

The cumulative incidence of overall post-transplant infection was 58.1% (95% CI: 54.5–61.7). In detail, 414 patients developed at least one episode of infection (incidence rate of 1.61 episodes per 1,000 transplant-days). The median interval from transplantation to the first episode was 57 days (IQR: 17.3–234.5). The cumulative incidence of the secondary outcomes of bacterial and opportunistic infection were 44.9% (95% CI: 41.3–48.6) and 22.8% (95% CI: 19.8–25.9), respectively.

### Unadjusted Analysis of Age and Post-Transplant Infection

**FIGURE 1:**
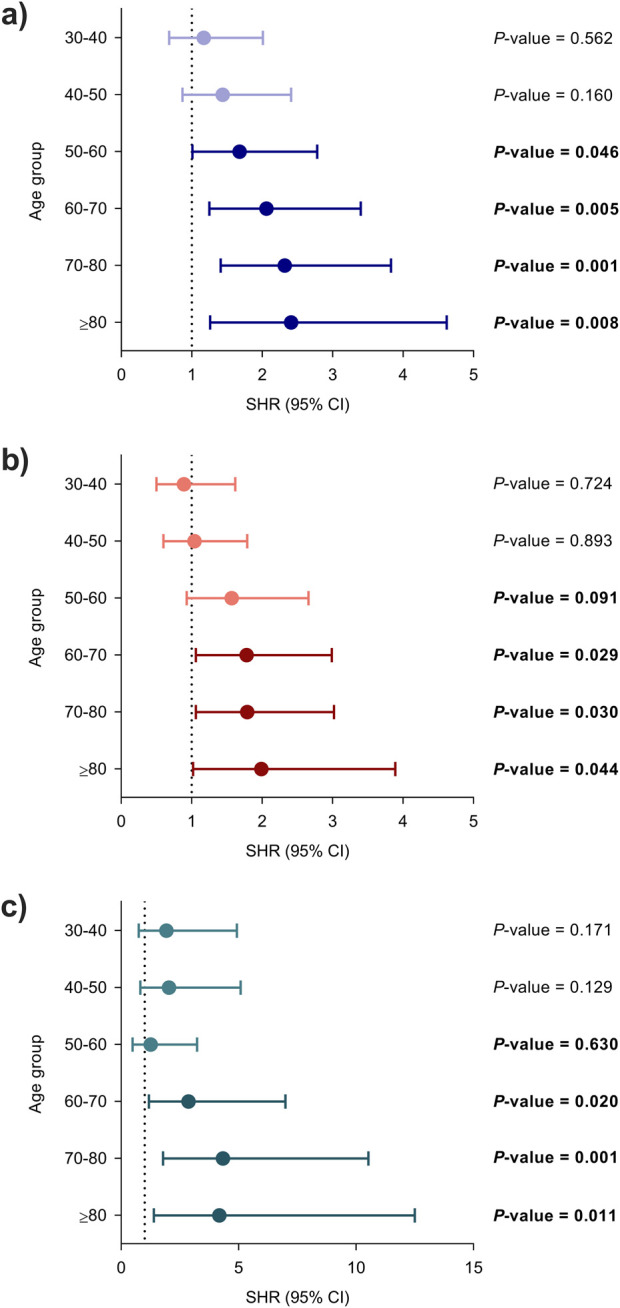
Unadjusted associations between recipient age at transplantation and the occurrence of infection using the lowest age group (<30 years) as the reference category: **(a)** overall, **(b)** bacterial and **(c)** opportunistic infection. Circles and bars represent the SHR and the limits of the 95% CI, respectively. CI, confidence interval; SHR, subdistribution hazard ratio.

First, we assessed whether recipient age exerted an effect on the risk of post-transplant infection in unadjusted analyses. Each 10-year increase was associated with the occurrence of overall infection (SHR: 1.18; 95% CI: 1.11–1.26; *P*-value < 0.001). Regarding secondary outcomes, the risks of bacterial (SHR: 1.17; 95% CI: 1.09–1.26; *P*-value < 0.001) and opportunistic infection (SHR: 1.26; 96% CI: 1.13–1.40; *P*-value < 0.001) were also increased with each 10-year increase. Using the lowest age group (<30 years) as the reference category, all the groups above 50 years had a significantly increased risk of overall infection, whereas the risk increase for bacterial and opportunistic infection achieved statistical significance for the age strata beyond 60 years (Figure 1).

**FIGURE 2:**
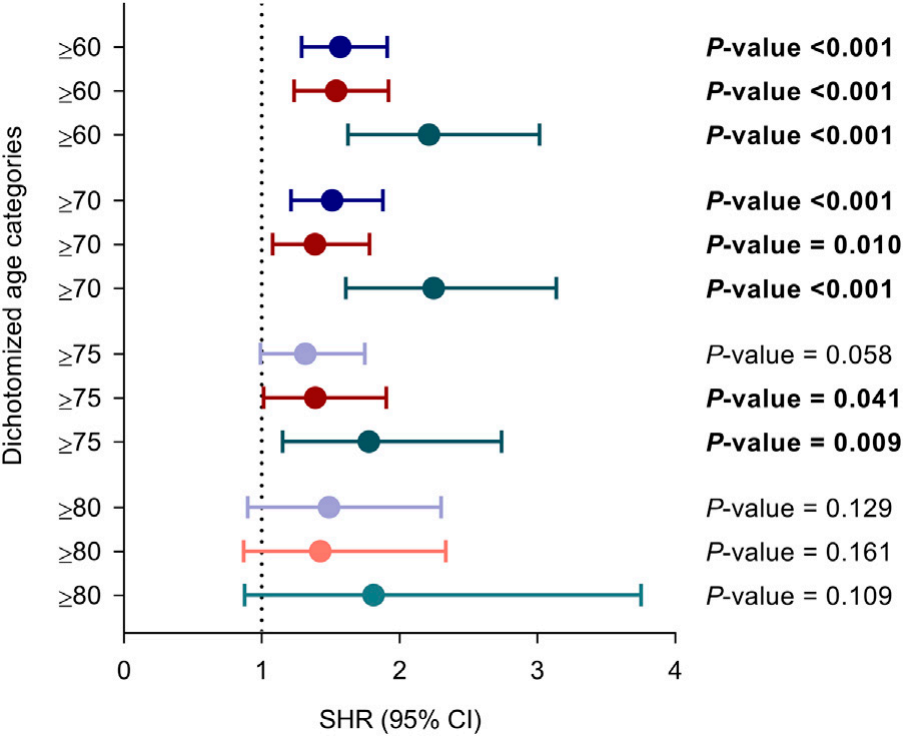
Unadjusted associations between recipient age at transplantation dichotomized according to different thresholds conventionally used to define “older adult KT recipient” and the occurrence of overall (purple), bacterial (red) and opportunistic infection (green). Circles and bars represent the SHR and the limits of the 95% CI, respectively. CI, confidence interval; SHR, subdistribution hazard ratio.

The variable was further dichotomized according to cut-off values conventionally used to define “older KT recipient”. Recipients ≥60 and ≥70 years have significantly increased risks for the primary and secondary outcomes as compared to those below these thresholds. For recipients ≥75 years these associations were only significant for the secondary outcomes, whereas none of the comparisons achieved statistical significance for the age category ≥80 years as compared to those below (Figure 2).

### Association Between Age and Post-Transplant Infection Adjusted for Clinical Covariables

As detailed in Supplementary Table S3, a number of variables were identified as predictive factors for the primary outcome of overall infection on the basis of their univariable *P*-values < 0.05. As expected, we found significant collinearity between receptor and donor age, between pre-transplant diabetes and diabetic nephropathy, and between living donor and cold ischemia time. Taking into account their clinical relevance, diabetes and living donor were kept in the model, in addition to recipient age. Therefore, the following covariates were included in the multivariable Fine and Gray’s regression model for the primary outcome of overall post-transplant infection: diabetes mellitus, chronic pulmonary disease, solid organ cancer, cerebrovascular disease, peripheral arterial disease, reflux nephropathy, CMV serology mismatch, pre-transplant dialysis, living donor, requirement of ICU admission, delayed graft function [DGF], early re-intervention and new-onset diabetes. After multivariable adjustment, each 10-year increase in recipient age continued to be independently associated with the occurrence of overall (adjusted SHR: 1.09; 95% CI: 1.02–1.18; *P*-value = 0.011).

Univariable analyses for the secondary outcomes are shown in Supplementary Tables S4, S5. After adjusting for the clinical covariates that achieved a *P*-value < 0.05 (diabetes mellitus, chronic pulmonary disease, cerebrovascular disease, peripheral arterial disease, reflux nephropathy, pre-transplant dialysis, type of donor, number of HLA mismatches, DGF, early re-intervention and new-onset diabetes), each 10-year increase in recipient age was independently associated with the occurrence of bacterial infection (adjusted SHR: 1.09; 95% CI: 1.00–1.18; *P*-value = 0.038). On the other hand, the association for opportunistic infection was not longer significant (adjusted SHR: 1.09; 95% CI: 0.96–1.25; *P*-value = 0.165) following multivariable adjustment (diabetes mellitus, coronary heart disease, reflux nephropathy, CMV serology mismatch, living donor, basiliximab induction, new-onset diabetes and acute graft rejection).

**FIGURE 3:**
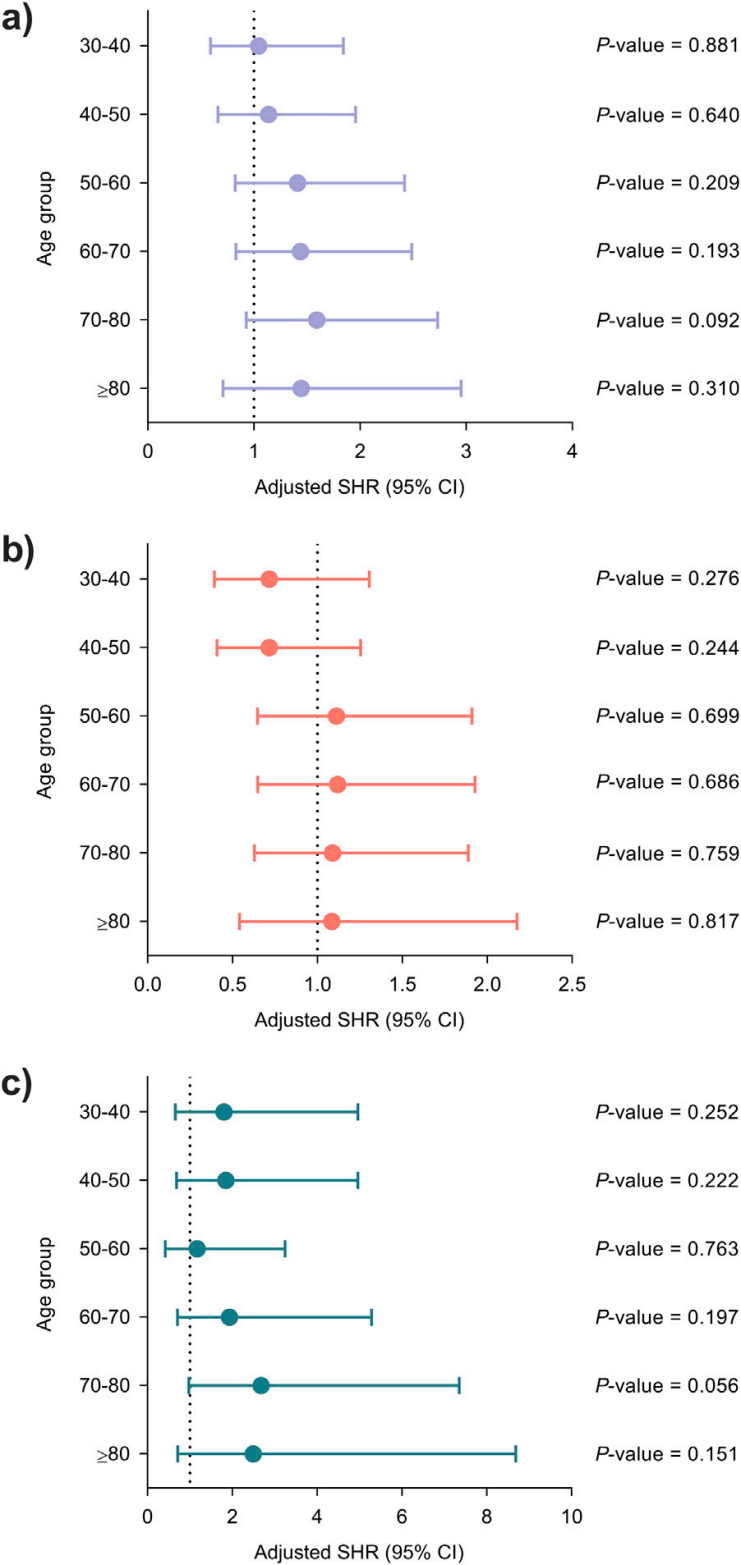
Adjusted associations between recipient age at transplantation and the occurrence of infection using the lowest age group (<30 years) as the reference category: **(a)** overall infection, **(b)** bacterial infection and **(c)** opportunistic infection. Models were adjusted for clinical factors with a *P-*value < 0.05 in the univariable analysis once ruled out the presence of significant collinearity, as detailed in Supplementary Tables S3–S5. Circles and bars represent the SHR and the limits of the 95% CI, respectively. CI, confidence interval; SHR, subdistribution hazard ratio.

There were no significant associations across increasing age groups with regards to the reference category (<30 years) for either the primary or secondary outcomes (Figure 3).

When age was dichotomized according to conventional thresholds, recipients ≥60 years exhibited a significantly higher risk of overall infection in the multivariable model (adjusted SHR: 1.25; 95% CI: 1.00–1.54; *P*-value = 0.045). As for secondary outcomes, the risk of opportunistic infection was significantly increased for recipients ≥70 years (adjusted SHR: 1.54; 95% CI: 1.02–2.32; *P*-value = 0.038) (Supplementary Figure S2).

### Immune Parameters According to Age Groups

**FIGURE 4:**
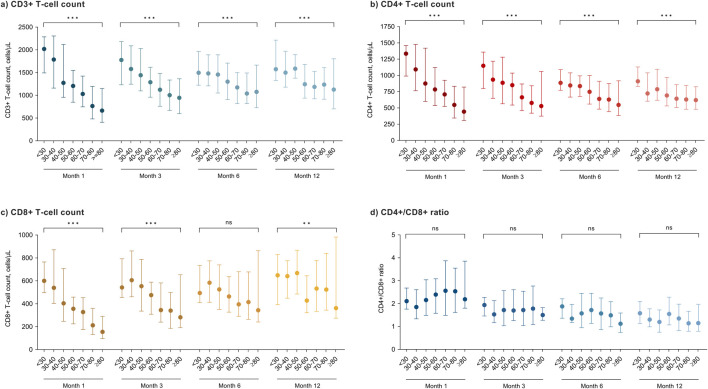
Peripheral blood lymphocyte subpopulation counts at different time points after transplantation according to age groups in KT recipients that did not receive T-cell-depleting induction therapy: **(a)** CD3^+^ T-cells; **(b)** CD4^+^ T-cells; **(c)** CD8^+^ T-cells and **(d)** CD4+/CD8+ ratio. Circles and bars represent the median and the interquartile range, respectively. ns, not significant; * *P*-value < 0.01; ** *P*-value < 0.001.

Finally, we compared across 10-year strata peripheral blood lymphocyte subpopulations counts assessed at different time points among KT recipients that did not receive ATG induction therapy (n = 371 patients with available data). There was a progressive decrease with increasing age for CD3^+^ and CD4^+^ T-cell counts at months 1, 3, 6 and 12 after transplantation *(P*-values for all comparisons <0.001). For the CD8^+^ T-cell count, the association was only significant at months 1 and 3 *(P*-values < 0.001), whereas the trend was less clear at month 12 *(P*-value = 0.008). No significant differences were found for the CD4+/C8+ ratio (Figure 4).

## Discussion

The present study aims to ascertain the relative contribution of recipient age to the susceptibility to infection after KT. We found that each 10-year increase was associated with an 18% increase in the risk of overall infection, and with a 17%–26% increase in the risks of bacterial and opportunistic infection. This gradient in the incidence of overall and bacterial infection remained significant after controlling for the confounding effect of chronic conditions, underling ESRD, pre-transplant dialysis, donor- and transplant-related factors (such as living donation or intraoperative transfusion requirements), and post-transplant complications (such as delayed graft function or re-intervention). Since the concept of “older adult recipient” is commonly operationalized by fixed thresholds, we also explored dichotomous cut-off values to found that recipients aged ≥60 years faced a significantly higher risk of infection, both at univariable and multivariable levels. In detail, this group experienced a 1.25-fold increase in the adjusted risk as compared to recipients younger than 60 years.

There is no clear consensus in the literature on the definition of older KT recipients, as the age limit for transplantation has been progressively expanded over the past decades. The increasingly accepted notion that age by itself should not be longer considered as an absolute contraindication must be empirically analyzed in light of graft and patient outcomes. For instance, Vanhove et al. [31] found that KT recipients >75 years had a four-fold higher absolute risk of DWFG as compared to those younger than 55 years, whereas a recent meta-analysis revealed a decreased survival beyond 70 years [23]. Infectious complications play a relevant role in these worse outcomes. In our experience with a cohort of KT recipients ≥75 years followed-up for more than 3 years, infection was the most common cause of death, far away from cardiovascular disease and *de novo* malignancy [10], and in line with other reported experiences [32].

Once established the independent effect on the infection risk of recipient age analyzed as continuous 10-year intervals, we attempted to discern whether there is a critical point above which the incidence rises exponentially. The results of the multivariable model suggest that this association is already evident for recipients ≥60 years. As graphically reflected by unadjusted and adjusted SHRs, the incidence of infection did not meaningfully change for patients aged 70–80 or ≥80 years as compared to lower strata (50–60 or 60–70), pointing to a plateau in the association between age and infection. In fact, the cut-off of 80 years was not discriminative for any of the outcomes analyzed. This result may be explained by the stringent selection process for KT candidates in older age group, which prioritizes patients with relatively low comorbidity burden and preserved functional status. In fact, the prevalence of relevant conditions such as diabetes mellitus or chronic pulmonary disease was lower in the group ≥80 years than in the previous age strata. On the other hand, the presence of CMV mismatch progressively decreased with age and was virtually absent beyond 80 years, mirroring the positive correlation between CMV seroprevalence and age observed in the general population [33]. Taking into account that most episodes of opportunistic infection consisted of CMV disease and that D+/R- mismatch is the strongest predictor for this complication, it is not completely unexpected the lack of significant association observed for octogenarians. These findings align with previous studies that reported similar patient survival [7] and comparable or even lower incidence of infection [34] among KT recipients ≥80 years as compared to those aged 70–79 years, in particular in the most recent era. Finally, it cannot be ruled out that the lack of significant associations observed for patients ≥80 years may be attributable to limited statistical power resulting from the small size of this group.

Despite the growing expansion of older KT candidates and recipients as a consequence of recent changes in kidney allocation policies, few studies have specifically investigated the relationship between recipient age and infection [35]. In a small single-center study that included 91 KT recipients ≥65 years (median age of 68 years), the incidence of urinary tract infection was found to be higher than in a control group aged 40–60 years matched by year of transplantation and gender. No differences were reported for other syndromes such as pneumonia or bloodstream infection [36]. Registry data from South Korea showed that the incidence of early post-transplant infection was increased in recipients ≥60 years, with the urinary tract as the most common site. In addition, this complication was associated to an increased risk of rejection, graft loss and all-cause mortality [37]. Unfortunately, no separate analyses by age strata were performed in these studies. In this context, a Swish study provides an age-stratified analysis of graft outcomes, showing that death with a functioning graft and graft loss increase with age [38]. Therefore, older age increases infection-related risks in KT recipients, contributing to death with a functioning graft and influencing graft loss. Another report from the United States Renal Data System database found an exponential rise in infection-related mortality across 10-year strata for KT recipients, in contrast to the linear increase observed for wait-listed patients. The mean age (43.6 years) and the study period (1988–1997) suggest that older recipients were underrepresented in this cohort [39].

Immunesenescence exerts profound effects on the cell-mediated adaptative immunity: decline in T-cell production, naïve/memory T-cell ratio imbalance, and reduction in the T-cell receptor repertoire diversity [15]. We observed an inverse biological gradient between age and CD3^+^, CD4^+^ and CD8^+^ T-cell counts in recipients that had not received ATG induction, particularly during the first months. These findings align with studies that demonstrated that older KT recipients experience an accelerated process of immunosenescence and exhibit an increase of senescent T-cell phenotypes [40, 41]. The clinical consequences are evident, since we and others have shown that low CD4^+^ and CD8^+^ T-cell counts are associated to a higher risk of infection after KT [42, 43]. The expansion of senescent and terminally differentiated T-cells with impaired capacity of response to novel antigens may contribute to the increased susceptibility to infection and the lower incidence of acute rejection observed in KT recipients of advanced age [40, 44, 45].

Newer strategies have been proposed to improve candidate selection for KT [46]. Once exclusively considered a clinical geriatric syndrome, frailty—which can be conceptualized as the age-related decline in the physiological reserve to stressors—has emerged as a key predictor of poor outcomes in transplant candidates and recipients [47, 48]. Frailty influences the susceptibility to and the severity of infection in non-transplant older patients [49, 50]. Interestingly, the prevalence of frailty among ESRD patients has been shown not to be directly correlated with biological age [47, 51]. Therefore, the assessment of frailty and functional status by means of validated tools [52] should be incorporated into the usual evaluation for inclusion on the waiting list and, presumably, in the infection risk stratification after KT.

Our study benefits from a large sample size, the granularity of clinical data (in contrast to registry-based reports [9, 37, 39]), the detailed assessment of the variable of interest, and the time-to-even analysis with death as the competing risk. Nevertheless, some limitations should be also acknowledged. As commented above, there is no established threshold for the definition of older adult KT recipient, with cut-off values ranging from 60 to 65 years in earlier studies [3, 53] to 80 years in more recent ones [7, 8]. As stated above, the numbers in some age groups were low, particularly for recipients ≥80 years, which may have limited the statistical power of certain comparisons. Geriatric functional assessment was not systematically performed although this evaluation is recommended for KT candidates by KDIGO guidelines, since frailty has been shown to act as a good marker of the risk of infection [54]. Finally, although our institutional protocols do not provide specific recommendations for the management of immunosuppression in older recipients, it is expected that the attending nephrologist had adjusted the target CNI trough levels according to the age of the patient and the functional status. Unfortunately, our database does not include such information.

Our study was ultimately aimed at establishing an empirical basis regarding the age threshold above which the infection risk disproportionately increases, thereby justifying some form of tailored intervention. In clinical practice, this might comprise a reduction of immunosuppression, such as the elective withdrawal of mycophenolic acid [55], the preferential use of steroid-sparing [56] or mTOR inhibitor-based regimens with reduced CNI exposure [57], or the tailored choice of induction therapy. Furthermore, it could be beneficial to include baseline lymphocyte counts in the evaluation of KT candidates to assess immunological status and individualize management strategies. The administration of primary or secondary prophylaxis against CMV for older R+ patients may be considered, eventually guided by some strategy of CMV-specific immune monitoring. In view of the association found with bacterial infection, another question is whether the use of antibiotic prophylaxis may provide some benefit for older recipients, a decision that should be carefully balanced against the risk of emergence of antimicrobial resistance.

In conclusion, after adjusting for numerous clinical covariates, we found a significant association between each 10-year increment in recipient age at KT and the occurrence of overall post-transplant infection. When clinically applicable thresholds were explored, this effect seems to be significant already for patients ≥60 years, whereas older groups would only marginally contribute to this risk increase. Further research is needed to refine the process of candidate selection by integrating functional assessments of frailty and to investigate individualized preventive interventions among susceptible older KT recipients.

## Data Availability

The datasets presented in this study can be found in online repositories. The names of the repository/repositories and accession number(s) can be found in the article/Supplementary Material.
