# Supplementary material for: Infection Risk in Older Kidney Transplant Recipients: An Analysis in the Era of Expanded Age Limits
Source: Transpl Int. 2025 Nov 12;38:15594. doi: 10.3389/ti.2025.15594 (PMC12646964; doi:10.3389/ti.2025.15594)

Fayos M, et al. *Infection risk in older kidney transplant recipients: an analysis in the era of expanded age limits.*

<u>Supporting Material</u>

Supplementary Methods

Additional study definitions

Cytomegalovirus (CMV) disease included viral syndrome and end-organ disease. Viral syndrome was defined by the presence of CMV DNAemia plus fever plus at least one of the following: leukopenia (white blood cell [WBC] count <3.50 x 10^3 cells/µL if baseline count was ≥4.00 x 10^3 cells/µL or a decrease >20% if baseline WBC count was <4.00 x 10^3 cells/µL); atypical lymphocytosis (≥5%); thrombocytopenia (platelet count <100 x 10^3 cells/µL if baseline count was ≥115 x 10^3 cells/µL or a decrease >20% if baseline platelet count was <115 x 10^3 cells/µL); or elevation of alanine amino transferase or aspartate amino transferase of more than 2 times the upper limit of normal. End-organ disease included probable or proven categories, with the latter requiring the documentation of CMV replication in tissue specimens by means of viral culture, immunohistochemistry, histopathology or DNA hybridization, in the presence of compatible clinical manifestations [1]. Presumptive BK polyomavirus-associated nephropathy was defined by the demonstration of plasma DNAemia >4 $\log_{10}$ copies/mL [2]. The diagnosis of *de novo* cancer had to be histologically confirmed. Delayed graft function was defined by the requirement of dialysis within the first week after transplantation. Acute graft rejection was suspected in case of sudden deterioration of graft function and confirmed by graft biopsy examination. Graft loss was defined by the definitive return to dialysis, nephrectomy and/or retransplantation.

References

1. Ljungman P, Boeckh M, Hirsch HH, Josephson F, Lundgren J, Nichols G, et al. Definitions of Cytomegalovirus Infection and Disease in Transplant Patients for Use in Clinical Trials. Clin Infect Dis. 2017;64:87-91.
2. Kotton CN, Kamar N, Wojciechowski D, Eder M, Hopfer H, Randhawa P, et al. The Second International Consensus Guidelines on the Management of BK Polyomavirus in Kidney Transplantation. Transplantation. 2024;108:1834-66.

Supplementary results

Table S1. Prevalence of pre-transplant comorbidities and transplant-related variables across different age groups.

**Variable**	**Age group** [n (%)]							*P*-value
	<30 years (n = 45)	30-40 years (n = 98)	40-50 years (n = 123)	50-60 years (n = 146)	60-70 years (n = 145)	70-80 years (n = 128)	≥80 years (n = 27)	
Pre-transplant comorbidities								
Hypertension	28 (62.2)	81 (82.7)	101 (82.1)	121 (82.9)	124 (85.5)	114 (89.1)	22 (81.5)	0.007
Diabetes mellitus	5 (11.1)	32 (32.7)	67 (54.5)	86 (58.9)	85 (58.6)	93 (72.7)	16 (59.3)	<0.001
Non-coronary heart disease	5 (11.1)	11 (11.2)	11 (8.9)	16 (11.0)	19 (13.1)	27 (21.1)	10 (37.0)	0.001
Coronary heart disease	0 (0.0)	4 (4.1)	7 (5.7)	11 (7.5)	30 (20.7)	22 (17.2)	5 (18.5)	<0.001
Chronic pulmonary disease	4 (8.9)	3 (3.1)	12 (9.8)	11 (7.5)	24 (16.6)	21 (16.4)	2 (7.4)	0.008
Solid organ cancer	2 (4.4)	4 (4.1)	4 (3.3)	12 (8.2)	16 (11.0)	26 (20.3)	7 (25.9)	<0.001
Cerebrovascular disease	0 (0.0)	2 (2.0)	3 (2.5)	7 (4.8)	9 (6.3)	20 (15.6)	5 (18.5)	<0.001
Peripheral arterial disease	0 (0.0)	1 (1.0)	5 (4.1)	9 (6.2)	18 (12.4)	11 (8.6)	3 (11.1)	0.004
Previous KT	8 (17.8)	14 (14.3)	28 (22.8)	22 (15.1)	23 (15.9)	11 (8.6)	1 (3.7)	0.043
D+/R- CMV serostatus	14 (31.3)	12 (12.2)	18 (14.6)	11 (7.5)	13 (9.0)	13 (10.2)	0 (0.0)	<0.001
Pre-transplant renal replacement therapy	33 (73.3)	86 (87.8)	109 (88.6)	124 (84.9)	130 (89.7)	119 (93.0)	25 (92.6)	0.026
DCD donor	6 (13.3)	14 (14.3)	31 (25.2)	42 (28.8)	20 (13.8)	11 (8.6)	2 (7.4)	<0.001
Living donor	18 (40.0)	30 (30.6)	17 (13.8)	29 (19.9)	15 (10.3)	7 (5.5)	0 (0.0)	<0.001

CMV: cytomegalovirus; D: donor; DCD: donation after circulatory death; KT: kidney transplantation; R: recipient.

Table S2. Description of clinical syndromes and causative agents of 1,088 episodes of post-transplant infection.

**Clinical syndrome**	**N (%)**
Urinary tract infection	340 (31.3)
Acute pyelonephritis	306 (28.1)
Perinephric abscess	23 (2.1)
Acute prostatitis	7 (6.4)
Orchitis	4 (0.4)
Pneumonia	91 (8.4)
Community-acquired pneumonia	73 (6.7)
Hospital-acquired pneumonia	15 (1.4)
Ventilator-associated pneumonia	3 (0.3)
Upper respiratory tract infection	95 (8.7)
Acute bronchitis	21 (1.9)
Digestive tract infection	114 (10.5)
Gastroenteritis	51 (4.7)
Clostridioides difficile infection	44 (4.0)
Colitis	14 (1.3)
Esophagitis	4 (0.4)
Ileitis	1 (1.0)
Viral syndrome	115 (10.5)
Skin and soft-tissue infection	90 (8.3)
Surgical site infection	62 (5.7)
Intraabdominal infection	32 (2.9)
Cholangitis	12 (1.1)
Secondary peritonitis	9 (0.8)
Spontaneous bacterial peritonitis	2 (0.2)
Cholecystitis	4 (0.4)
Liver abscess	3 (0.3)
Diverticulitis	1 (0.1)
Intraabdominal abscess	1 (0.1)
Catheter-related bacteremia	21 (1.9)
Septic thrombophlebitis	5 (0.5)
Endocarditis	3 (0.3)
Sinusitis and other ENT infections	16 (1.5)
BK polyomavirus-associated nephropathy	6 (0.6)
Meningitis	2 (0.2)
Bone and joint infection	9 (0.9)
Other	66 (6.1)
**Isolated microorganisms**	**N (%)**
Bacteria	764 (70.2)
Gram-negative bacilli	343 (31.5)

Escherichia coli	136 (12.5)
Klebsiella pneumoniae	92 (8.5)
Klebsiella oxytoca	8 (7.4)
Enterobacter spp.	17 (1.6)
Campylobacter spp.	11 (1.0)
Citrobacter spp.	5 (4.6)
Proteus spp.	10 (0.9)
Salmonella spp.	2 (0.2)
Other Enterobacterales	3 (0.3)
Pseudomonas aeruginosa	55 (5.1)
Other	4 (0.4)
Gram-positive cocci	81 (7.4)
Enterococcus faecalis	30 (2.8)
Enterococcus faecium	20 (1.8)
Staphylococcus aureus	15 (1.4)
Coagulase-negative staphylococci	10 (0.9)
Streptococcus pneumoniae	2 (0.2)
Nocardia spp.	2 (0.2)
Other	2 (0.2)
Anaerobes	47 (4.3)
Clostridioides difficile	44 (4.0)
Polymicrobial infection	3 (0.3)
Mycobacterium tuberculosis complex	1 (0.1)
Treponema pallidum	1 (0.1)
No microbiological diagnosis[a]	291 (26.7)
Viruses	285 (26.2)
CMV	102 (9.4)
HSV-1 and 2	27 (2.5)
Varicella-zoster virus	34 (3.1)
BK polyomavirus	12 (1.1)
Respiratory viruses	106 (9.7)
SARS-CoV-2	55 (5.1)
Influenza virus	38 (3.5)
Respiratory syncytial virus	9 (0.8)
Human metapneumovirus	2 (0.2)
Rhinovirus	2 (0.2)
Norovirus	1 (0.1)
Rotavirus	1 (0.1)
Parvovirus B19	1 (0.1)
Monkeypox	1 (0.1)
Fungi	35 (3.2)
Yeasts	29 (2.7)
Candida spp.	26 (2.4)
Cryptococcus neoformans	1 (0.1)

Trichosporon spp.	1 (0.1)
Malassezia furfur	1 (0.1)
Filamentous fungi	4 (0.4)
Aspergillus spp.	2 (0.2)
Rhizopus spp.	1 (0.1)
Exophiala oligospermun	1 (0.1)
Pneumocystis jirovecii	2 (0.2)
Parasites	4 (0.4)
Cryptosporidum spp.	2 (0.2)
Strogyloides stercoralis	1 (0.1)
Giardia lamblia	1 (0.1)

[a] Episodes with clinical response to empirical antibiotic treatment and no microbiological documentation.

CMV: cytomegalovirus; ENT: ear, nose and throat; HSV: herpes simplex virus; SARS-CoV-2: severe acute respiratory syndrome coronavirus 2.

Table S3. Univariable analysis of factors predictive for overall post-transplant infection (primary study outcome).

Variable	No infection (n = 298)	Infection (n = 414)	*P*-value
Male gender [n (%)]	193 (65.0)	283 (68.4)	0.346
BMI at transplantation, Kg/m^2 [mean ± SD]	25.6 ± 8.6	25.9 ± 4.3	0.651
Prior or current smoking history [n (%)]	102 (34.2)	167 (40.3)	0.097
Pre-transplant conditions [n (%)]			
Hypertension	249 (83.6)	342 (82.6)	0.740
Diabetes mellitus	67 (22.5)	137 (33.1)	**0.002**
Non-coronary chronic heart disease	41 (13.8)	58 (14.0)	0.924
Coronary heart disease	26 (8.7)	53 (12.8)	0.087
Chronic pulmonary disease	23 (7.7)	54 (13.0)	**0.024**
Solid organ cancer	22 (7.4)	49 (11.8)	**0.050**
Cerebrovascular disease	12 (4.0)	34 (8.3)	**0.024**
Lower limb peripheral arterial disease	10 (3.4)	37 (8.9)	**0.003**
Previous solid organ transplantation [n (%)]	53 (17.9)	63 (15.3)	0.361
Underlying end-stage kidney disease [n (%)]			
Glomerulonephritis	65 (21.8)	86 (20.8)	0.738
Diabetic nephropathy	47 (15.8)	94 (22.7)	**0.022**
Polycystic kidney disease	44 (14.8)	54 (13.0)	0.511
Nephroangiosclerosis	26 (8.7)	43 (10.4)	0.460
Chronic interstitial nephropathy	13 (4.4)	22 (5.3)	0.562
Loss of renal mass and hyperfiltration injury	7 (2.3)	12 (2.9)	0.645
Congenital nephropathy	17 (5.7)	13 (3.1)	0.093
Reflux nephropathy	16 (5.4)	6 (1.4)	**0.003**
Lupus nephropathy	10 (3.4)	15 (3.6)	0.848

Unknown	32 (10.7)	46 (11.1)	0.875
Other	21 (7.0)	23 (5.6)	0.784
CMV serostatus [n (%)]			
R+	262 (87.9)	350 (84.5)	0.201
D+/R-	24 (8.1)	57 (13.8)	**0.018**
D-/R-	12 (4.0)	7 (1.7)	0.056
Positive EBV serostatus (anti-EBNA IgG) [n (%)]	284 (95.3)	391 (94.4)	0.611
Positive HCV serostatus [n (%)]	15 (5.1)	33 (8.0)	0.132
Positive HBsAg status [n (%)]	8 (2.7)	12 (2.9)	0.871
Positive HIV serostatus [n (%)]	1 (0.3)	4 (1.0)	0.406
Pre-transplant renal replacement therapy [n (%)]	247 (82.9)	379 (91.5)	**<0.001**
Hemodialysis	193 / 247 (78.1)	306 / 379 (80.7)	0.429
Continuous ambulatory peritoneal dialysis	54 / 247 (21.9)	73 / 379 (19.3)	
Time on dialysis, days [median (IQR)]	667.5 (373.3 – 1,368.8)	720.5 (376.3 – 1,382)	0.459
Age of donor, years [mean ± SD]	50.9 ± 16.5	56.1 ± 16.7	**<0.001**
Type of donor [n (%)]			
DBD donor	181 (60.7)	289 (69.8)	**0.012**
DCD donor	46 (15.4)	80 (19.3)	0.180
Living donor	71 (23.8)	45 (10.9)	**<0.001**
Cold ischemia time, hours [median (IQR)]	14.7 ± 7.8	16.4 ± 7.3	**0.002**
Number of HLA mismatches [median (IQR)]	4 (3 – 5)	4 (3 – 5)	0.087
Intraoperative blood product transfusion [n (%)]	23 (7.7)	51 (12.3)	**0.047**
Requirement of ICU admission [n (%)][a]	1 (0.3)	11 (2.7)	**0.018**
Induction therapy [n (%)]			
Antithymocyte globulin	132 (44.3)	175 (42.3)	0.590
Basiliximab	132 (44.3)	191 (46.1)	0.627
None	34 (11.4)	48 (11.6)	0.939
Primary immunosuppression [n (%)]			0.797

Prednisone, tacrolimus and MMF/MPS	280 (94.3)	387 (93.5)	
Prednisone, tacrolimus and azathioprine	12 (4.0)	17 (4.1)	
Prednisone, tacrolimus and mTOR inhibitor	5 (1.5)	10 (2.4)	
CMV antiviral prophylaxis [n (%)]	156 (52.5)	219 (53.0)	0.895
Post-transplant complications [n (%)]			
Delayed graft function	86 (28.9)	204 (49.3)	**<0.001**
Surgical re-intervention[b]	22 (7.4)	54 (13.0)	**0.016**
New-onset diabetes	28 (9.4)	69 (16.7)	**0.005**
Renal artery stenosis	30 (10.1)	59 (14.3)	0.096
De novo DSA development	14 (4.7)	30 (7.3)	0.160
Biopsy-proven acute graft rejection	21 (7.0)	46 (11.1)	0.067
Acute rejection during the first month	11 (3.7)	12 (2.9)	0.555
Acute rejection during the first 3 months	14 (4.7)	22 (5.3)	0.711

BMI: body mass index; CMV: cytomegalovirus; D: donor; DBD: donation after brain death; DCD: donation after circulatory death; DSA: donor-specific antibody; EBV: Epstein-Barr virus; HCV: hepatitis C virus; HBsAg: hepatitis B virus surface antigen; HIV: human immunodeficiency virus; HLA: human leukocyte antigen; ICU: intensive care unit; IQR: interquartile range; MPA: enteric-coated mycophenolate sodium; MMF: mycophenolate mofetil; mTOR: mammalian target of rapamycin; SD: standard deviation; R: recipient.

[a] Within the first two weeks after transplantation.

[b] Within the first month after transplantation.

Table S4. Univariable analysis of factors predictive for post-transplant bacterial infection (secondary study outcome).

**Variable**	**No bacterial infection** (n = 392)	**Bacterial infection** (n = 320)	***P*-value**
Gender of recipient (male) [n (%)]	259 (66.2)	217 (67.8)	0.658
BMI at transplantation, Kg/m^2 [median (IQR)]	25.7 ± 7.8	25.8 ± 4.4	0.865
Prior or current smoking history [n (%)]	139 (35.5)	130 (40.6)	0.157
Pre-transplant conditions [n (%)]			
Hypertension	331 (84.4)	260 (81.3)	0.260
Diabetes mellitus	90 (23.0)	114 (35.6)	**<0.001**
Non-coronary chronic heart disease	49 (12.5)	50 (15.6)	0.231
Coronary heart disease	39 (9.9)	40 (12.5)	0.281
Chronic pulmonary disease	31 (7.9)	46 (14.4)	**0.006**
Solid organ cancer	38 (9.7)	33 (10.3)	0.784
Cerebrovascular disease	18 (4.6)	28 (8.8)	**0.026**
Lower limb peripheral arterial disease	14 (3.6)	33 (10.3)	**<0.001**
Previous solid organ transplantation [n (%)]	69 (17.7)	47 (14.8)	0.306
Underlying end-stage kidney disease [n (%)]			
Glomerulonephritis	85 (21.7)	66 (20.6)	0.731
Diabetic nephropathy	66 (16.8)	75 (23.4)	**0.028**
Polycystic kidney disease	55 (14.0)	43 (13.4)	0.819
Nephroangiosclerosis	39 (9.9)	30 (9.4)	0.797
Chronic interstitial nephropathy	19 (4.8)	16 (5.0)	0.925
Loss of renal mass and hyperfiltration injury	9 (2.3)	10 (3.2)	0.495
Congenital nephropathy	19 (4.8)	11 (3.4)	0.352
Reflux nephropathy	18 (4.6)	4 (1.3)	**0.010**
Lupus nephropathy	13 (3.3)	12 (3.8)	0.754

Unknown	45 (11.5)	33 (10.3)	0.620
Other	24 (6.1)	20 (6.3)	0.943
CMV serostatus [n (%)]			
R+	332 (84.7)	280 (87.5)	0.284
D+/R-	48 (12.2)	33 (10.3)	0.419
D-/R-	12 (3.1)	7 (2.2)	0.472
Positive EBV serostatus (anti-EBNA IgG) [n (%)]	371 (94.6)	304 (95.0)	0.831
Positive HCV serostatus [n (%)]	22 (5.7)	26 (8.2)	0.192
Positive HBsAg status [n (%)]	9 (2.3)	11 (3.4)	0.362
Positive HIV serostatus [n (%)]	3 (0.8)	2 (0.6)	1.000
Pre-transplant renal replacement therapy [n (%)]	330 (84.2)	296 (92.5)	**<0.001**
Hemodialysis	255 / 330 (77.3)	244 / 296 (82.4)	0.109
Continuous ambulatory peritoneal dialysis	75 / 330 (22.7)	52 / 296 (17.6)	
Time on dialysis, days [median (IQR)]	712 (383 – 1,436)	699 (371 – 1,333)	0.820
Age of donor, years [mean ± SD]	52.5 ± 16.7	55.7 ± 16.7	**0.010**
Cold ischemia time, hours [median (IQR)]	15.1 ± 7.8	16.5 ± 7.2	**0.019**
Type of donor [n (%)]			
DBD donor	256 (65.3)	214 (66.9)	0.660
DCD donor	53 (13.5)	73 (22.8)	**0.001**
Living donor	83 (21.2)	33 (10.3)	**<0.001**
Number of HLA mismatches [mean ± SD]	4 (3 – 5)	4 (3 – 5)	**0.049**
Intraoperative blood product transfusion [n (%)]	39 (9.9)	35 (10.9)	0.667
Requirement of ICU admission [n (%)][a]	4 (1.0)	8 (2.5)	0.127
Induction therapy [n (%)]			
Antithymocyte globulin	163 (41.6)	144 (45.0)	0.360
Basiliximab	184 (46.9)	139 (43.4)	0.351
None	45 (11.5)	37 (11.6)	0.973
Primary immunosuppression [n (%)]			0.732

Prednisone, tacrolimus and MMF/MPS	365 (93.4)	302 (94.4)	
Prednisone, tacrolimus and azathioprine	18 (4.6)	11 (3.4)	
Prednisone, tacrolimus and mTOR inhibitor	8 (2.0)	7 (2.2)	
CMV antiviral prophylaxis [n (%)]	202 (51.7)	173 (54.2)	0.495
Post-transplant complications [n (%)]			
Delayed graft function	124 (31.6)	166 (51.9)	**<0.001**
Surgical re-intervention[b]	30 (7.7)	46 (14.4)	**0.004**
New-onset diabetes	43 (11.0)	54 (16.9)	**0.022**
Renal artery stenosis	41 (10.5)	48 (15.0)	0.068
De novo DSA development	23 (5.9)	21 (6.6)	0.684
Biopsy-proven acute graft rejection	31 (7.9)	36 (11.3)	0.129
Acute rejection during the first month	14 (3.6)	9 (2.8)	0.569
Acute rejection during the first 3 months	21 (5.4)	15 (4.7)	0.685

BMI: body mass index; CMV: cytomegalovirus; D: donor; DBD: donation after brain death; DCD: donation after circulatory death; DSA: donor-specific antibody; EBV: Epstein-Barr virus; HCV: hepatitis C virus; HBsAg: hepatitis B virus surface antigen; HIV: human immunodeficiency virus; HLA: human leukocyte antigen; ICU; intensive care unit; IQR: interquartile range; MPA: enteric-coated mycophenolate sodium; MMF: mycophenolate mofetil; mTOR: mammalian target of rapamycin; SD: standard deviation; R: recipient.

[a] Within the first two weeks after transplantation.

[b] Within the first month after transplantation.

Table S5. Univariable analysis of factors predictive for post-transplant opportunistic infection (secondary study outcome).

**Variable**	**No opportunistic infection** (n = 550)	**Opportunistic infection** (n = 162)	***P*-value**
Gender of recipient (male) [n (%)]	361 (65.8)	115 (71.0)	0.214
BMI at transplantation, Kg/m^2 [median (IQR)]	25.7 ± 6.9	26.2 ± 4.5	0.428
Prior or current smoking history [n (%)]	202 (36.7)	67 (41.4)	0.285
Pre-transplant conditions [n (%)]			
Hypertension	455 (82.7)	136 (84.0)	0.716
Diabetes mellitus	144 (26.2)	60 (37.0)	**0.007**
Non-coronary chronic heart disease	77 (14.0)	22 (13.6)	0.892
Coronary heart disease	51 (9.3)	28 (17.3)	**0.004**
Chronic pulmonary disease	57 (10.4)	20 (12.3)	0.475
Solid organ cancer	49 (8.9)	22 (13.6)	0.081
Cerebrovascular disease	31 (5.7)	15 (9.4)	0.093
Lower limb peripheral arterial disease	32 (5.8)	15 (9.3)	0.121
Previous solid organ transplantation [n (%)]	90 (16.5)	26 (16.0)	0.889
Underlying end-stage kidney disease [n (%)]			
Glomerulonephritis	121 (22.0)	30 (18.5)	0.341
Diabetic nephropathy	102 (18.5)	39 (24.1)	0.121
Polycystic kidney disease	80 (14.5)	18 (11.1)	0.265
Nephroangiosclerosis	48 (8.7)	21 (13.0)	0.109
Chronic interstitial nephropathy	26 (4.7)	9 (5.6)	0.668
Loss of renal mass and hyperfiltration injury	16 (2.9)	3 (1.9)	0.463
Congenital nephropathy	25 (4.5)	5 (3.1)	0.417
Reflux nephropathy	21 (3.8)	1 (0.6)	**0.039**
Lupus nephropathy	20 (3.6)	5 (3.1)	0.738

Unknown	55 (10.0)	23 (14.2)	0.133
Other	36 (6.5)	8 (4.9)	0.513
CMV serostatus [n (%)]			
R+	482 (87.6)	130 (80.2)	**0.017**
D+/R-	49 (8.9)	32 (19.8)	**<0.001**
D-/R-	19 (3.5)	0 (0.0)	0.011
Positive EBV serostatus (anti-EBNA IgG) [n (%)]	524 (95.3)	151 (93.2)	0.298
Positive HCV serostatus [n (%)]	37 (6.8)	11 (6.8)	1.000
Positive HBsAg status [n (%)]	17 (3.1)	3 (1.9)	0.589
Positive HIV serostatus [n (%)]	3 (0.5)	2 (1.2)	0.320
Pre-transplant renal replacement therapy [n (%)]	476 (86.5)	150 (92.6)	**0.038**
Hemodialysis	380 / 476 (79.8)	119 / 150 (79.3)	0.895
Continuous ambulatory peritoneal dialysis	96 / 476 (20.2)	31 / 150 (20.7)	
Time on dialysis, days [median (IQR)]	702 (367 – 1,394.8)	715.5 (407 – 1,321)	0.769
Age of donor, years [mean ± SD]	52.1 ± 16.5	60.0 ± 16.3	**<0.001**
Cold ischemia time, hours [median (IQR)]	15.4 ± 7.7	16.9 ± 6.9	**0.026**
Type of donor [n (%)]			
DBD donor	344 (62.5)	126 (77.8)	**<0.001**
DCD donor	103 (18.7)	23 (14.2)	0.184
Living donor	103 (18.7)	13 (8.0)	**<0.001**
Number of HLA mismatches [mean ± SD]	4 (3 – 5)	5 (3 – 5)	0.212
Intraoperative blood product transfusion [n (%)]	48 (8.7)	26 (16.0)	**0.007**
Requirement of ICU admission [n (%)][a]	7 (1.3)	5 (3.1)	0.157
Induction therapy [n (%)]			
Antithymocyte globulin	246 (44.7)	61 (37.7)	0.110
Basiliximab	236 (42.9)	87 (53.7)	**0.015**
None	68 (12.4)	14 (8.6)	0.192
Primary immunosuppression [n (%)]			0.332

Prednisone, tacrolimus and MMF/MPS	519 (94.5)	148 (91.4)	
Prednisone, tacrolimus and azathioprine	20 (3.6)	9 (5.6)	
Prednisone, tacrolimus and mTOR inhibitor	10 (1.8)	5 (3.1)	
CMV antiviral prophylaxis [n (%)]	298 (54.3)	77 (47.8)	0.149
Post-transplant complications [n (%)]			
Delayed graft function	216 (39.3)	74 (45.7)	0.145
Surgical re-intervention[b]	54 (9.8)	22 (13.6)	0.173
New-onset diabetes	67 (12.2)	30 (18.5)	**0.039**
Renal artery stenosis	64 (11.6)	25 (15.4)	0.199
De novo DSA development	30 (5.5)	14 (8.8)	0.131
Biopsy-proven acute graft rejection	43 (7.8)	24 (14.8)	**0.007**
Acute rejection during the first month	13 (2.4)	10 (6.2)	**0.016**
Acute rejection during the first 3 months	21 (3.8)	15 (9.3)	**0.005**

BMI: body mass index; CMV: cytomegalovirus; D: donor; DBD: donation after brain death; DCD: donation after circulatory death; DSA: donor-specific antibody; EBV: Epstein-Barr virus; HCV: hepatitis C virus; HBsAg: hepatitis B virus surface antigen; HIV: human immunodeficiency virus; HLA: human leukocyte antigen; ICU; intensive care unit; IQR: interquartile range; MPA: enteric-coated mycophenolate sodium; MMF: mycophenolate mofetil; mTOR: mammalian target of rapamycin; SD: standard deviation; R: recipient.

[a] Within the first two weeks after transplantation.

[b] Within the first month after transplantation.

Figure S1. Species distribution of 285 microbiologically documented episodes of post-transplant urinary tract infection.

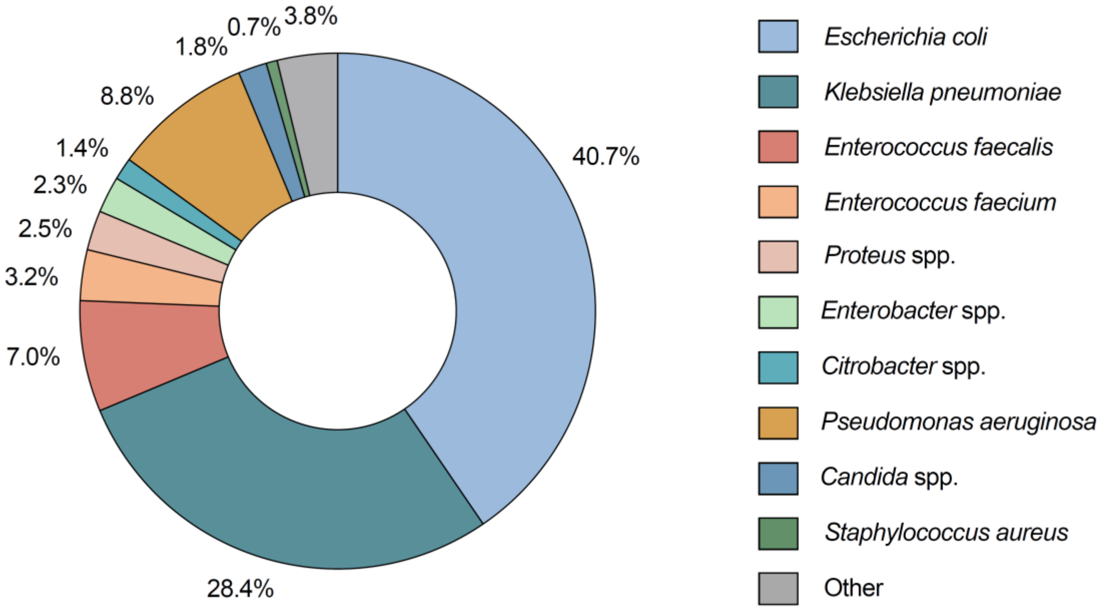

Figure S2. Adjusted associations between recipient age at transplantation dichotomized according to different thresholds and the occurrence of overall (purple), bacterial (red) and opportunistic infection (green). Circles and bars represent the SHR and the limits of the 95% CI, respectively. CI: confidence interval; SHR: subdistribution hazard ratio.

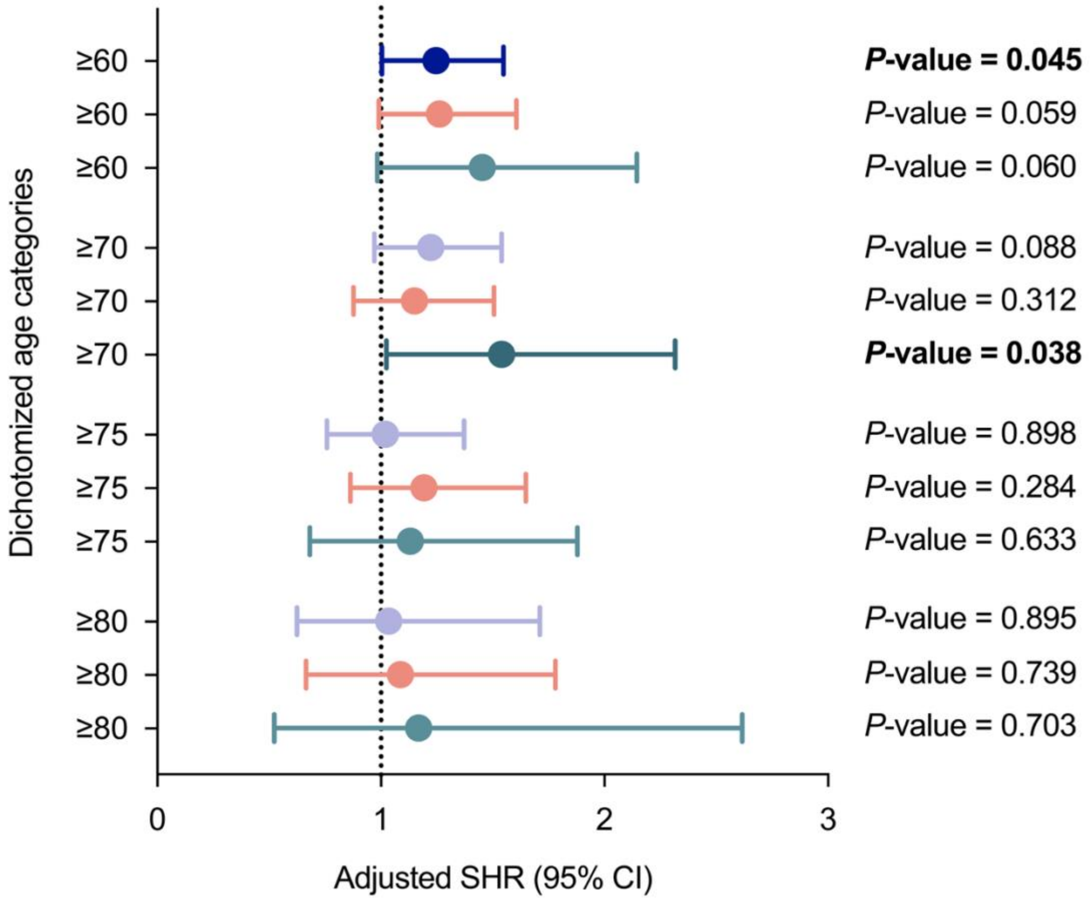

Supplement: Supplementary file 1 [file DataSheet1.pdf]
